# Alveolar pentraxin 3 as an early marker of microbiologically confirmed pneumonia: a threshold-finding prospective observational study

**DOI:** 10.1186/s13054-014-0562-5

**Published:** 2014-10-15

**Authors:** Tommaso Mauri, Andrea Coppadoro, Michela Bombino, Giacomo Bellani, Vanessa Zambelli, Carla Fornari, Lorenzo Berra, Edward A Bittner, Ulrich Schmidt, Marina Sironi, Barbara Bottazzi, Paolo Brambilla, Alberto Mantovani, Antonio Pesenti

**Affiliations:** Department of Health Science, University of Milan-Bicocca, Via Cadore 48, 20900 Monza, Italy; Department of Emergency Medicine, San Gerardo Hospital, Via Pergolesi 33, 20900 Monza, Italy; Research Centre on Public Health, Department of Statistics and Quantitative Methods, University of Milan-Bicocca, Via Pergolesi 33, 20900 Monza, Italy; Department of Anesthesia, Critical Care, and Pain Medicine, Massachusetts General Hospital and Harvard Medical School, 55 Fruit St, 02114 Boston, MA USA; Department of Inflammation and Immunology, Humanitas Clinical and Research Center, Via Manzoni 56, 20089 Rozzano, MI Italy; Department of Translational Medicine, University of Milan, Via Festa del Perdono 7, 20122 Rozzano, MI Italy

## Abstract

**Introduction:**

Timely diagnosis of pneumonia in intubated critically ill patients is rather challenging. Pentraxin 3 (PTX3) is an acute-phase mediator produced by various cell types in the lungs. Animal studies have shown that, during pneumonia, PTX3 participates in fine-tuning of inflammation (for example, microbial clearance and recruitment of neutrophils). We previously described an association between alveolar PTX3 and lung infection in a small group of intubated patients. The aim of the present study was to determine a threshold level of alveolar PTX3 with elevated sensitivity and specificity for microbiologically confirmed pneumonia.

**Methods:**

We recruited 82 intubated patients from two intensive care units (San Gerardo Hospital, Monza, Italy, and Massachusetts General Hospital, Boston, MA, USA) undergoing bronchoalveolar lavage (BAL) as per clinical decision. We collected BAL fluid and plasma samples, together with relevant clinical and microbiological data. We assayed PTX3 and soluble triggering receptor expressed on myeloid cells 1 (sTREM-1) in BAL fluid and PTX3, sTREM-1, C-reactive protein (CRP) and procalcitonin (PCT) in plasma. Two blinded independent physicians reviewed patient data to confirm pneumonia. We determined the PTX3 threshold in BAL fluid for pneumonia and compared it to other biomarkers.

**Results:**

Microbiologically confirmed pneumonia of bacterial (*n* =12), viral (*n* =4) or fungal (*n* =8) etiology was diagnosed in 24 patients (29%). PTX3 levels in BAL fluid predicted pneumonia with an area under the receiving operator curve of 0.815 (95% CI =0.710 to 0.921, *P* <0.0001), whereas none of the other biomarkers were effective. In particular, PTX3 levels ≥1 ng/ml in BAL fluid predicted pneumonia in univariate analysis (β =2.784, SE =0.792, *P* <0.001) with elevated sensitivity (92%), specificity (60%) and negative predictive value (95%). Net reclassification index PTX3 values ≥1 ng/ml in BAL fluid for pneumonia indicated gain in sensitivity and/or specificity vs. all other mediators. These results did not change when we limited our analyses only to confirmed cases of bacterial pneumonia. Moreover, when we considered only the 70 patients who fulfilled the clinical criteria for the diagnosis of pneumonia at BAL fluid sampling, the diagnostic accuracy of PTX levels was confirmed in univariate and ROC curve analysis.

**Conclusions:**

In this hypothesis-generating convenience sample, a PTX3 level ≥1 ng/ml in BAL fluid was discriminative of microbiologically confirmed pneumonia in mechanically ventilated patients.

**Electronic supplementary material:**

The online version of this article (doi:10.1186/s13054-014-0562-5) contains supplementary material, which is available to authorized users.

## Introduction

Timely and accurate diagnosis of pneumonia is a challenging task for intensive care unit (ICU) physicians [1]. According to the Centers for Disease Control and Prevention guidelines, the clinical diagnosis is made on the basis of the presence of fever, leukocytosis, purulent lung secretions and new infiltrates seen on chest X-rays [2]. Microbiological culture of bronchoalveolar lavage (BAL) fluid represents an accepted standard to confirm (or exclude) a clinical diagnosis of pneumonia in intubated ICU patients [3], but it generally takes 48 hours to obtain results [4]. Meanwhile, physicians usually prescribe either broad-spectrum antibiotics [5] or probability-based, narrow-spectrum antibiotics [6]. In fact, both approaches yield many false-positive pneumonia diagnoses, resulting in these patients being given unnecessary antibiotics until culture results become available [7]. Thus, extensive research efforts have been spent in recent years to accelerate rapid and accurate diagnosis of pneumonia in ICU patients [8-11]. For example, the Clinical Pulmonary Infection Score (CPIS) has proved helpful in diagnosing ventilator-associated pneumonia (VAP) [9]. Measuring circulating procalcitonin (PCT) levels has been shown to be effective in guiding antibiotic therapy in critically ill patients with suspected bacterial infection without adverse events [10]. In another study, researchers proposed soluble triggering receptor expressed on myeloid cells 1 (sTREM-1) in BAL fluid as an early biomarker for the diagnosis of pneumonia in intubated patients [8]. However, CPIS has been validated only for making a VAP diagnosis, and PCT and sTREM-1 results have been challenged [11-14]. To our knowledge, an accurate and reliable early marker for pneumonia in intubated ICU patients is still lacking.

Pentraxin 3 (PTX3) is an acute-phase inflammatory mediator produced at the site of infection that can be assayed in a few hours [15]. In the lungs, epithelial cells, endothelial cells and leukocytes can produce PTX3 if appropriately stimulated [15-17]. During experimental pneumonia, PTX3 has been shown to recognize different infectious agents (that is, bacteria, viruses and fungi) and to enhance their clearance, primarily by regulation of neutrophil recruitment [18-20]. In humans, plasma PTX3 levels correlate with clinical severity in many infectious diseases [21-24]. Circulating PTX3 is elevated in the most severe forms of VAP and community-acquired pneumonia (CAP) [25,26], and pleural fluid PTX3 measurement improves discrimination of parapneumonic effusions [27]. We previously described an association between detectable alveolar PTX3 levels and lung infections of any etiology in a small group of intubated patients with acute respiratory distress syndrome (ARDS) [28].

Given the sound pathophysiological background and the experimental data in animal models and patients, we conducted the present two-center international study to identify the threshold PTX3 level in BAL fluid for accurate diagnosis of microbiologically confirmed pneumonia and compare the diagnostic accuracy of alveolar PTX3 levels with those of other biomarkers.

## Methods

### Study population

We performed a prospective, threshold-finding observational study with patients admitted to two university-affiliated ICUs (San Gerardo Hospital, Monza, Italy, and Massachusetts General Hospital, Boston, MA, USA). The inclusion criteria were age ≥18 years, intubation and mechanical ventilation and BAL fluid obtained as per clinical decision. The exclusion criteria were insufficient quantity of recovered BAL fluid, BAL performed in a patient previously enrolled in the same study, organizational reasons (for example, temporary unavailability of the research team) and refusal to give consent. The ethics committee of the San Gerardo Hospital and the Partners Human Research Committee of the Massachusetts General Hospital approved the research protocol. Informed consent was obtained either from the patients or, when deemed incompetent to provide consent, from their next of kin.

### Bronchoalveolar lavage fluid collection and analysis

BAL fluid was obtained using a standard technique [28]. Briefly, a fiberscope was aseptically introduced through the endotracheal tube to the most peripheral bronchus of the radiographically positive lobe (that is, where new pulmonary infiltrates seemed evident). Next, fixed aliquots (*n* =6, 20 ml each) of 0.9% sterile saline solution were instilled into the aforementioned lobe, and each aliquot was gently aspirated. Each BAL procedure took 10 to 20 minutes to perform. The first recovered BAL fluid portion was discarded. The remainder was mixed and sent in part to the microbiology department (for bacterial quantitative cultures as appropriate; for viruses (*Aspergillus fumigatus*, *Candida* spp and *Pneumocystis carinii*), cultures were obtained only when clinically suspected (that is, in immunocompromised patients)) and in part to the cytology department for semiquantitative leukocyte counts (ranging from no leukocytes (−) to few (+) or many (++ or +++)). The remaining BAL fluid was centrifuged, and the supernatant was stored at −80°C, along with one plasma sample. Within 1 month after fluid and plasma collection, biological samples were transferred in dry ice to the Department of Inflammation and Immunology, Humanitas Clinical and Research Center, Rozzano (MI), Italy.

Upon arrival of the fluid samples, biomarkers were assayed in duplicate using the following procedures. (1) In one experiment, plasma and alveolar PTX3 was assayed with a specific enzyme-linked immunosorbent assay (ELISA) kit developed in-house (intra- and interassay coefficients of variation <10%, sensitivity of 0.1 ng/ml, assay dynamic range from 75 pg/ml to 2.4 ng/ml). Out-of-range BAL fluid samples were diluted at a 1:2 to 1:18 ratio and plasma specimens at a 1:5 to 1:90 ratio. This assay does not cross-react with C-reactive protein (CRP) or serum amyloid A protein. The time required to perform a single measurement was about 6 to 7 hours. The cost to perform one measurement was €5 per patient [28]. (2) Plasma and BAL fluid sTREM-1 levels were measured using a commercially available sandwich ELISA kit (Sigma-Aldrich, St Louis, MO, USA) following the manufacturer instructions (detection limit 8 pg/ml). We also performed in-house validation measurements (see Additional file 1). (3) We measured plasma CRP levels by turbidimetric immunoassay (Roche/Hitachi Modular PRE-ANALYTICS system, P module; Roche Diagnostics, Milan, Italy). (4) Plasma PCT levels were measured using commercially available kits (Elecsys BRAHMS PCT Assay; Roche Diagnostics, Mannheim, Germany), with a detection limit of 0.02 ng/ml. On the same day as BAL was performed, we recorded patients’ comorbidities and several parameters of disease severity.

### Diagnosis of pneumonia

Two experienced intensivists blinded to biomarker levels and to the initial clinical diagnoses independently reviewed BAL cultures and patients’ medical records to classify each case as CAP (defined as clinical suspicion of pneumonia arising <48 hours after hospital admission), healthcare-associated pneumonia (HCAP, defined as clinical suspicion of pneumonia arising <48 hours after hospital admission in a patient recently hospitalized and/or living in a nursing home or long-term care facility and/or who received parenteral antimicrobial therapy within 30 days before diagnosis of pneumonia), hospital-acquired pneumonia (HAP, defined as clinical suspicion arising ≥48 hours after hospital admission and intubation initiated <48 hours after hospital admission), VAP (clinical suspicion arising ≥48 hours after hospital admission and intubation performed ≥48 hours after hospital admission) or no pneumonia [2,6,8]. The pneumonia diagnosis was based upon the following standard criteria [29]:

1. New and persistent radiographic infiltrates associated with at least two of the following:
  - Internal body temperature >38°C,
  - White blood cells count >12,000 or <4,000 cells/mm^3^ and/or
  - Purulent tracheobronchial secretions; as well as with:
2. A positive BAL culture (that is, noncontaminant bacteria or fungi identified in 10^4^ or more colony-forming units per milliliter and/or significant noncontaminant viral load).

Pneumonia cases of fungal and viral etiology were carefully reviewed and then confirmed if risk factors were present (for example, immunosuppression), airway colonization was excluded (for example, presence of systemic infection signs, contemporary presence of other biofluids infected by the same pathogen, exclusion of other infection sites) and specific antifungal or antiviral therapy was started.

Pneumonia was considered absent when clinical signs of pneumonia were not present at the time of BAL fluid sampling and/or when an alternative cause for pulmonary infiltrate was established and there was no significant growth in BAL cultures and/or colonization, together with absence or full recovery from clinical signs of sepsis without any or new antibiotic therapy.

### Immunostaining to measure cell fractions positive for intracellular PTX3

The cell pellets of 20 BAL fluid aliquots (24%) from 20 consecutive patients (all enrolled in Monza, Italy) were resuspended, spun and stained to measure the fraction of cells in BAL fluid positive for intracellular PTX3, as previously described [30].

### Statistical analysis

Statistical analysis was performed using SAS 9.2 software (SAS Institute, Cary, NC, USA). A *P*-value <0.05 was considered statistically significant. The percentage agreement and Cohen’s κ were calculated to compare the performance of the two independent intensivists’ assessments of the pneumonia diagnoses [31]. Normally distributed variables (Shapiro-Wilk test) are reported as mean ± standard deviation, and non-normal variables as median and interquartile range (IQR). Comparisons of quantitative variables were carried out using Student’s *t*-test or the Mann–Whitney *U* test as appropriate. For comparison of categorical variables, a χ^2^ test or Fisher’s exact test (in cases of small cell sizes, expected values <5) was performed. The correlation coefficient between biomarkers was analyzed using Spearman’s ρ coefficient.

The discriminatory ability of each biomarker was measured as the area under the receiving operating characteristic curve (AUC^ROC^) with pneumonia as the outcome of interest. AUC^ROC^ values with 95% CIs and *P*-values for differences from chance are reported. Sensitivity, specificity, positive predictive value (PPV), negative predictive value (NPV) and Youden index [32] values were computed to evaluate the PTX3 thresholds in BAL fluid and plasma with highest discriminatory ability for pneumonia. Univariate logistic regression was used to determine predictors of pneumonia. β-values (that is, log odds ratio), standard errors (SEs) and *P*-values for differences from 0 (the Wald statistic) are reported. The net reclassification index (NRI) was used to compare the performance of the identified cutoff PTX3 levels in BAL fluid vs. other biomarkers and CPIS; that is, we used the cutoff identified by the Youden index value for plasma PTX3 and clinically accepted cutoffs for all other mediators and CPIS. More details on the methods used in this study can be found in Additional file 1.

## Results

### Study population

**Figure 1:**
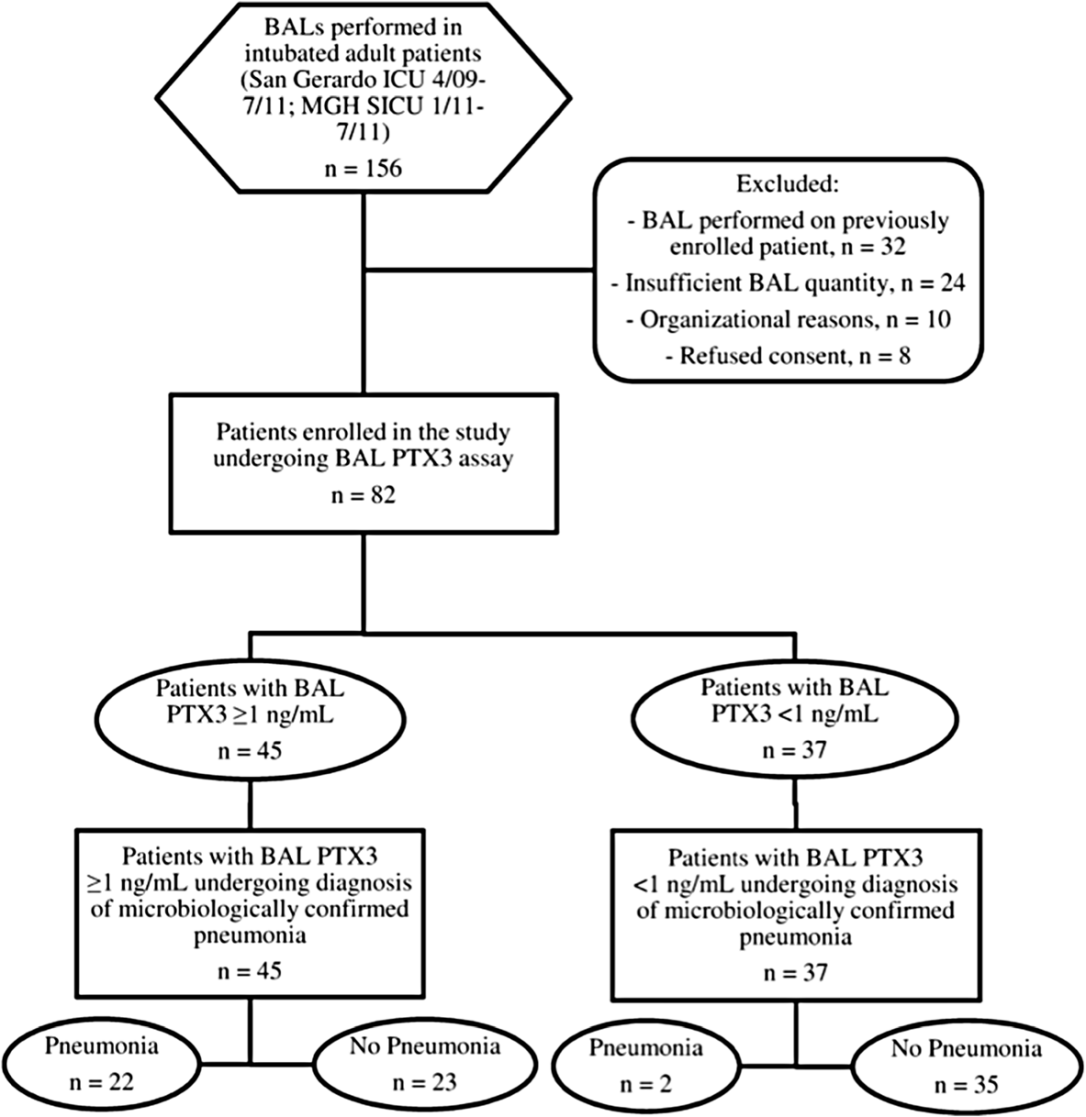
**Flow diagram for the study cohort.** BAL, Bronchoalveolar lavage; ICU, Intensive care unit; MGH, Massachusetts General Hospital; PTX, Pentraxin 3; SICU, Surgical intensive care unit.

We enrolled a convenience sample of 82 patients, including 64 in Monza between April 2009 and July 2011 and 18 in Boston between January and July 2011. Figure 1 presents a flow diagram for the study population. The reasons reported by the attending physicians for performing BAL in these patients were suspected lung infection (*n* =66; 80%), suspected aspiration pneumonia (*n* =3; 4%), suspected malignancies (*n* =4; 5%), suspected autoimmune disease (*n* =4; 5%), exclusion of significant bacterial growth to start steroids (*n* =2; 2%) and undefined (*n* =3; 4%). The main characteristics of the patients are listed in Table 1. Among the 82 enrolled patients, 23 were female (28%), mean patient age was 59 ± 17 years, mean Simplified Acute Physiology Score II at the time of ICU admission was 46 ± 15 and in-hospital mortality was 30% (*n* =25).

**Table 1:** **Main characteristics at the time of bronchoalveolar lavage fluid and plasma sampling in the study cohort** ^**a**^

**Characteristics**	**All BAL** **(** ***N*** **= 82)**	**Pneumonia** **(** ***n*** **= 24, 29%)**	**No pneumonia** **(** ***n*** **= 58, 71%)**
Days since hospital admission	8 (4 to 16)	5.5 (2.5 to 10.5)	9 (6 to 17)
Comorbidities, *n* (%)			
Hypertension	21 (26)	6 (25)	15 (26)
Ischemic vascular disease	14 (17)	5 (21)	9 (16)
Diabetes	9 (11)	2 (8)	7 (12)
COPD	12 (15)	4 (17)	8 (14)
Malignancy	16 (20)	6 (25)	10 (17)
Chronic kidney disease	6 (7)	2 (8)	4 (7)
Other	10 (12)	3 (12)	7 (12)
Days since intubation	2 (1 to 7)	2 (0.5 to 6.5)	2.5 (1 to 7)
FiO_2_, %	50 (40 to 70)	57.5 (40 to 62.5)	50 (40 to 70)
PaO_2_/FiO_2_ ratio, mmHg	188.6 (138.0 to 232.0)	198.4 (144.3 to 249.9)	178.2 (136.7 to 220)
PEEP, cmH_2_O	10 (8 to 12)	10 (8 to 12)	10 (8 to 12)
V_t_, ml/kg	6.6 ± 2.2	7.4 ± 1.9	6.3 ± 2.2
Body temperature, °C	37.9 (36.9 to 38.5)	37.6 (36.7 to 38.4)	38.0 (37.0 to 38.50)
White blood cells, 10^3^/mm^3^	11.5 (8.7 to 15.9)	13.4 (7.7 to 17.3)	11.3 (8.9 to 14.9)
Heart rate, beats/min	90.6 ± 18.2	96.6 ± 15.6	88.1 ± 18.8
SOFA score	6 (4 to 8)	7 (4.5 to 10)	6 (4 to 8)
Organ failures, *n*	2 (1 to 2)	2 (1 to 3)	2 (1 to 2)
Septic shock, *n* (%)	28 (34)	9 (38)	19 (33)
Immunosuppression, *n* (%)	20 (24)	8 (33)	12 (21)
Receiving antibiotics, *n* (%)	73 (89)	21 (88)	52 (90)
CPIS	3 (2 to 5)	4 (2.5 to 5)	3 (2 to 5)
Plasma PCT, ng/ml	1.0 (0.3 to 6.1)	2.1 (0.4 to 8.2)	0.9 (0.3 to 4.0)
Plasma CRP, mg/dl	13.9 (7.5 to 22.7)	9.5 (6.0 to 21.7)	14.4 (7.7 to 23.6)
Plasma sTREM-1, pg/ml	346.0 (216.0 to 685.0)	345.0 (230.5 to 779.5)	352.0 (216.0 to 620.0)
Plasma PTX3, ng/ml	56.2 (31.4 to 127.4)	62.7 (31.0 to 174.1)	54.3 (31.4 to 110.5)
BAL fluid sTREM-1, pg/ml	0 (0 to 281)	42.5 (0 to 316)	0 (0 to 247)
BAL fluid PTX3, ng/ml	1.2 (0.3 to 7.4)	8.3 (1.6 to 29.5)^b^	0.6 (0.0 to 3.4)
Semiquantitative BAL fluid leukocyte count, *n* (%)			
−	10 (12)	1 (4)	9 (15)
+	25 (30)	4 (17)	21 (36)
++	24 (30)	11 (46)	13 (22)
+++	23 (28)	8 (33)	15 (26)

^a^−, No leukocytes; +, Few leukocytes; ++ and +++, Many leukocytes; BAL, Bronchoalveolar lavage; COPD, Chronic obstructive pulmonary disease; CPIS, Clinical Pulmonary Infection Score; CRP, C-reactive protein; FiO_2_, Fraction of inspired oxygen; PaO_2_, Partial pressure of oxygen in arterial blood; PCT, Procalcitonin; PEEP, Positive end-expiratory pressure; PTX3, Pentraxin 3; SOFA, Sequential Organ Failure Assessment; sTREM-1, Soluble triggering receptor expressed on myeloid cells 1; V_t_, Tidal volume. Mean ± standard deviation values are indicated for normally distributed variables, and median (interquartile range) values are given for non-normal variables. ^b^ *P* < 0.0001 vs. no pneumonia.

### Identification of bronchoalveolar lavage fluid PTX3 threshold for microbiologically confirmed pneumonia diagnosis

Among patients enrolled in the study, data review by two independent physicians blinded to biomarker levels and the initial diagnosis yielded coincident results in 79 cases (agreement rate =0.963, Cohen’s κ =0.911). The three controversial cases were then jointly reviewed, and consensus was achieved in all of them. Lung infection was diagnosed in 24 cases (29%); among these, 14 were VAP (58%), 7 were HAP (30%) and 3 were CAP (12%). No HCAP case was identified. The etiology of pneumonia was bacterial in 12 cases (50%), fungal in 8 (33%) and viral in 4 (25%) (see Table E1 in Additional file 1). When BAL was performed, clinical criteria for the diagnosis of pneumonia were lacking in 12 cases (15%).

**Figure 2:**
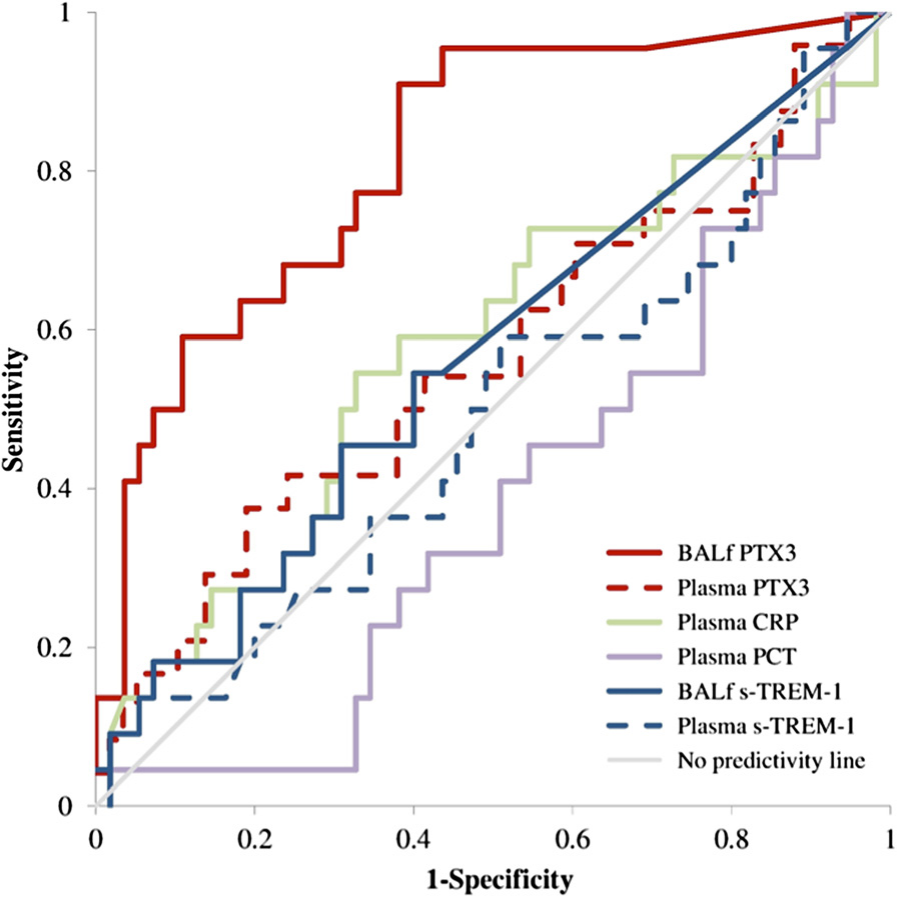
**Pentraxin 3 as an early marker of pneumonia.** Area under the receiver operating characteristic curve (AUC^ROC^) analysis showed that Pentraxin 3 (PTX3) levels in bronchoalveolar lavage fluid (BALf) levels predicted pneumonia (AUC^ROC^ =0.815, 95% CI =0.710 to 0.921, *P* <0.0001), but that BALf levels of soluble triggering receptor expressed on myeloid cells 1 (s-TREM-1), plasma PTX3, C-reactive protein (CRP) and procalcitonin (PCT) levels did not. A cutoff of PTX3 levels ≥1 ng/ml in BAL fluid (identified by Youden index) was associated with 92% sensitivity, 60% specificity, 49% positive predictive value and 95% negative predictive value for culture-positive pneumonia.

PTX3 levels in BAL fluid was the only mediator significantly higher in the presence of pneumonia compared to noninfection cases (*P* <0.0001) (Table 1). AUC^ROC^ analysis showed that PTX3 levels in BAL fluid predicted pneumonia (AUC^ROC^ =0.815, 95% CI =0.710 to 0.921, *P* <0.0001), whereas BAL fluid levels of sTREM-1, plasma PTX3, sTREM-1, CRP and PCT did not (Figure 2). A cutoff level of PTX3 ≥ 1 ng/ml in BAL fluid (identified by Youden index [32]) was associated with 92% sensitivity, 60% specificity, 49% PPV and 95% NPV for culture-positive pneumonia. Total accuracy was 70%. Of the two patients with pneumonia and PTX3 level <1 ng/ml in BAL fluid, one had bacterial CAP and the other had fungal HAP and immunosuppression (which could have determined low PTX3 levels). Among the 23 patients without pneumonia and with PTX3 levels ≥1 ng/ml in BAL fluid, 5 had cardiogenic pulmonary edema, 9 had ARDS of extrapulmonary origin, 4 had primary noninfectious ARDS and 2 had acute exacerbations of chronic obstructive pulmonary disease (all representing acute inflammatory conditions of lung that can elevate unspecific biomarkers like PTX3), and a final diagnosis could not be determined in 3 patients.

Net reclassification indexes for PTX3 levels ≥1 ng/ml in BAL fluid were elevated (range =0.38 to 0.55) in comparison to all other mediators and to CPIS with gain in sensitivity, specificity or both (Table 2). In univariate prediction analysis, PTX3 levels ≥1 ng/ml in BAL fluid significantly predicted pneumonia (β =2.784, SE =0.792, *P* <0.001); all other biomarkers were not effective (Table 3).

**Table 2:** **Comparison of net reclassification index values for PTX3 levels in bronchoalveolar lavage fluid** ^**a**^

**Comparisons with BAL fluid PTX3** ≥**1 ng/ml**	**Δ** **Se**	**Δ** **(1-SP)**	**NRI**
Plasma PTX3 ≥130 ng/ml	0.58	−0.21	0.38
Plasma PCT ≥0.5 ng/ml	0.25	0.19	0.44
Plasma CRP ≥0.5 mg/dl	−0.09	0.61	0.52
BAL fluid sTREM-1 ≥5 pg/ml	0.38	0.07	0.44
Plasma sTREM-1 ≥5 pg/ml	−0.04	0.60	0.56
CPIS ≥7	0.88	−0.34	0.53

^a^BAL, Bronchoalveolar lavage fluid; CPIS, Clinical Pulmonary Infection Score; CRP, C-reactive protein; NRI, Net reclassification index; PCT, Procalcitonin; PTX3, Pentraxin 3; ΔSe, Change in sensitivity; Δ(1-SP), Change in 1-specificity; sTREM-1, Soluble triggering receptor expressed on myeloid cells 1.

**Table 3:** **Univariate analysis for prediction of microbiologically confirmed pneumonia** ^**a**^

**Variables**	**Univariate β**	**SE**	***P*** **-value**
BAL fluid PTX3 ≥1 ng/ml	2.784	0.792	<0.001
Plasma PTX3 ≥130 ng/ml	0.742	0.576	0.198
Plasma PCT ≥0.5 ng/ml	0.506	0.532	0.342
Plasma CRP ≥0.5 mg/dl^b^	–	–	–
BAL fluid sTREM-1 ≥5 pg/ml	0.438	0.507	0.389
Plasma sTREM-1 ≥5 pg/ml^c^	–	–	–
CPIS ≥7	−0.192	1.183	0.871

^a^BAL, Bronchoalveolar lavage fluid; CPIS, Clinical Pulmonary Infection Score; CRP, C-reactive protein; PCT, Procalcitonin; PTX3, Pentraxin 3; SE, standard error; sTREM-1, Soluble triggering receptor expressed on myeloid cells 1. ^b^Could not be determined because all values were >0.5 mg/dl. ^c^Could not be determined, as estimates were not reliable because of convergence problems of the statistical model.

### Bronchoalveolar lavage fluid PTX3 levels in patients with confirmed bacterial pneumonia

When we compared the 58 negative cases to the 12 patients with proven bacterial pneumonia (total *N* =70), PTX3 diagnostic accuracy was confirmed by AUC-ROC analysis (AUC^ROC^ =0.827, 95% CI =0.668 to 0.987, *P* <0.0001); however, BAL fluid sTREM-1, plasma PTX3, sTREM-1, CRP and PCT levels were not predictive of bacterial pneumonia (see Figure E1 in Additional file 1). A new cutoff level of PTX3 levels ≥7 ng/ml in BAL fluid (identified by Youden index [32]) was associated with 75% sensitivity, 88% specificity, 56% PPV and 94% NPV for confirmed bacterial pneumonia. Total accuracy was 70%. Net reclassification indexes for PTX3 levels ≥7 ng/ml in BAL fluid were elevated (range =0.32 to 0.66) in comparison to all other mediators and to CPIS with gains in sensitivity, specificity or both. In univariate prediction analysis, PTX3 levels ≥7 ng/ml in BAL fluid significantly predicted pneumonia (β =2.931, SE =0.768, *P* <0.001), whereas all other mediators could not predict bacterial pneumonia.

### Bronchoalveolar lavage fluid PTX3 levels in patients who fulfilled clinical criteria for pneumonia

When we considered only the 70 patients who fulfilled clinical criteria for the diagnosis of pneumonia at the time of BAL fluid sampling, PTX3 diagnostic accuracy was confirmed both by both the AUC^ROC^ curve (AUC^ROC^ =0.838, 95% CI =0.737 to 0.941, *P* <0.0001) and univariate analysis (PTX3 levels ≥1 ng/ml in BAL fluid: β =3.065, SE =0.809, *P* <0.001), whereas all other mediators could not predict pneumonia.

### Results of immunostaining to measure cell fractions positive for intracellular PTX3

**Figure 3:**
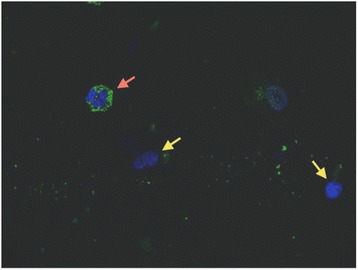
**Pentraxin 3 is stored in alveolar cell granules.** Immunostained images show intracellular presence of Pentraxin 3 (PTX3) inside one cell (red arrow) recovered from the alveolar space of an intubated critically ill patient. In the present study, cells recovered from 20 consecutive bronchoalveolar lavage (BAL) procedures in 20 intubated critically ill patients (see the immunostaining paragraph in the Methods section for details) were stained to measure the fraction of cells remaining after BAL that were positive for intracellular PTX3. The shape of the cell in this picture resembles that of an alveolar leukocyte, which constitutively stores PTX3 inside specific granules. Green represents a fluorescent anti-human PTX3 antibody, and blue are is the cell nucleus stained with bisbenzimide. Yellow arrows indicate nuclei of BAL cells that, apparently, don't store PTX3.

Cells retrieved by BAL with intracellular PTX3 were present in all immunostained samples (*n* =20, all from the Monza cohort), and the median percentage of cells in BAL fluid positive for intracellular PTX3 was 36% (IQR =32% to 51%) (Figure 3). PTX3 levels in BAL fluid levels and percentages of cells in BAL fluid positive for intracellular PTX3 were not correlated (*P* >0.05; data not shown). More results can be found in Additional file 1.

## Discussion

### Main findings

In a convenience sample of intubated critically ill patients undergoing BAL as per clinical decision, alveolar PTX3 was an early marker of microbiologically confirmed pneumonia with better diagnostic accuracy than other biomarkers. The diagnostic accuracy of PTX3 was confirmed when we limited our analyses only to proven bacterial pneumonia cases or to patients who fulfilled clinical criteria for pneumonia at the time of BAL fluid sampling.

### Bronchoalveolar lavage fluid PTX3 levels and diagnosis of pneumonia

Pneumonia represents one of the most widespread infections in intubated ICU patients [6] and is associated with significant morbidity (for example, prolonged mechanical ventilation, end-stage organ failure), costs and mortality [6]. Timely and accurate diagnosis of pneumonia is crucial to starting appropriate treatment and improving patients’ recovery and survival. However, to date, a rapid and accurate approach to diagnose pneumonia in intubated critically ill patients is lacking [2]. The aim of the present study was to describe the use of PTX3 levels in BAL fluid as a potentially new marker for early and accurate diagnosis of pneumonia. The study design was observational in one population of unselected intubated critically ill patients. Our results indicate that rapid assay of PTX3 levels in BAL fluid can predict the presence of lung infection about 48 hours before culture data become available [4]. To help with translation to the clinical setting, we identified a cutoff level of 1 ng/ml as being associated with the highest sum of sensitivity and specificity [32], and we found that this level was associated with high diagnostic accuracy. In particular, PTX3 levels ≥1 ng/ml in BAL fluid showed high sensitivity and NPV. This indicates that PTX3 levels ≥1 ng/ml in BAL fluid only rarely misclassify a patient with pneumonia as being uninfected. This is probably the most clinically relevant result we can report, as recognition of true negative cases is crucial for antibiotics stewardship aimed at reducing microbial resistance [6]. The same results were confirmed when we performed a *post hoc* analysis of proven bacterial pneumonia cases, with a higher threshold (7 ng/ml) and, possibly, improved specificity. Moreover, when we restricted our analysis to the subgroup of patients who met clinical criteria for pneumonia at the time of BAL, our results were also confirmed. In this subset of patients, pretest probability was higher and the diagnostic accuracy of a screening test such as PTX3 was expected to be poorer. Another strength of the study is that oxygenation did not differ between infected and uninfected patients. We previously showed that plasma PTX3 level is correlated with the severity of ARDS [28], but this did not seem to bias the present results. Our data thus lead us to the working hypothesis that PTX3 level in BAL fluid is discriminative of confirmed pneumonia in intubated ICU patients. In the future, adequately powered prospective trials may validate PTX3 level measured in BAL fluid assays as a marker of pneumonia and test whether it has any clinical value in guiding prescription of antibiotics.

### Comparative diagnostic accuracy

In our study, alveolar PTX3 was an independent marker of pneumonia due to any etiology with elevated NRI values in comparison to other mediators and to CPIS. In 2004, in an observational single-center study, Gibot and colleagues proposed sTREM-1 as an early and accurate marker of pneumonia [8]. However, none of the patients enrolled in their study presented with viral pneumonia [8]. In two more recent studies, researchers found that sTREM-1 levels in BAL fluid are not discriminative for pneumonia in intubated ICU patients [12,13]. Large validation studies on the role of plasma PCT in reducing antibiotics exposure yielded encouraging results [10]. Our results on the association between circulating PCT and pneumonia diagnosis, at variance, are poor. There are various possible reasons for this apparent contrast. First, in the PCT arm of the PRORATA trial, antibiotics were prescribed and stopped based on variations in PCT levels, thus implying an association between the trend of PCT levels in time and infection [10]. Second, a lack of association between the PCT value in one sample and pneumonia diagnosis has also been shown by other recent studies [33]. Third, PCT is known to have no diagnostic value in pneumonia of nonbacterial origin [10]. CPIS is a clinical test for screening VAP cases in intubated critically ill patients [9]. However, in our population, CPIS yielded poor results in predicting pneumonia. To this end, one should consider that CPIS has been validated only in VAP and that 56% of our sample were non-VAP cases. A possibly higher diagnostic accuracy of PTX3 levels in BAL fluid in comparison to other biomarkers was confirmed in the *post hoc* analysis of proven bacterial pneumonia cases.

Thus, our study indicates that, in intubated critically ill patients, the accuracy of PTX3 levels in BAL fluid for diagnosing pneumonia might be superior to other current biomarkers and clinical markers, especially when nonbacterial and/or non-VAP cases are suspected. Still, the diagnostic value of plasma PTX3 might be markedly increased by serial measurements over time (for example, at 24 to 48 hours after BAL) [22], which could be tested in future studies.

### Correlation between intra- and extracellular alveolar PTX3

Previous studies showed that neutrophils, which constitutively store PTX3 in specific granules, rapidly release PTX3 in response to microorganisms and many other inflammatory stimuli [18-20,28]. In our population, we found a high prevalence of cells in BAL fluid that stained positive for intracellular PTX3 by anti-hPTX3 rabbit antibodies [30], which may be the result of new recruitment of fresh leukocytes in response to the infection. In our patients, PTX3 levels in BAL fluid were not correlated with the percentage of intracellular PTX3-positive cells. Besides the relevance of leukocyte activation in the lungs of mechanically ventilated ICU patients who may be infected [34], other factors may play a role in determining PTX3 levels in BAL fluid. Rapid induction of PTX3 expression has been described in a variety of different cell types and as being involved in host local response during lung infections: monocytes, macrophages, alveolar epithelial and endothelial cells, and fibroblasts [21]. Thus, the lack of association between intra- and extracellular alveolar PTX3 may be due to the combination of *de novo* synthesis of the protein by immune and stromal cells and release of the stored protein from leukocytes.

### Study limitations

This study has many limitations, and therefore the information regarding alveolar PTX3 should be interpreted with caution. (1) We conducted an observational, hypothesis-generating study with a relatively modest sample size, and the clinical impact of PTX3 levels in BAL fluid-based protocols for the diagnosis of pneumonia and for prescribing (or withholding) of antibiotics remains to be determined in larger, prospective randomized studies. (2) The timing of BAL was not preplanned; instead, we collected BAL fluid whenever the attending physician decided to perform BAL, to the point that 15% of total cases did not fulfill the clinical criteria for pneumonia. In this way, we could evaluate PTX3 diagnostic performance in real-life clinical routine. Moreover, all results were confirmed in the subgroup of cases with clinical criteria for suspected pneumonia. (3) The PTX3 assay used in this study has a relatively narrow dynamic range, thus requiring dilution of biological samples with higher concentrations. This may limit the clinical robustness of the results, as translation into routine practice is far easier with tests involving undiluted samples. However, 60% of the BAL fluid samples, which were the main focus of this study, were tested without any dilution, as they were within the assay dynamic range. Nonetheless, more effort should be put into development of new PTX3 assays with wider dynamic ranges to help bench-to-bedside translation. (4) Immunostaining was performed in only 20 BAL fluid samples (24%); however, the immunostained samples were consecutive and pneumonia incidence and PTX3 levels in BAL fluid in this subgroup closely resembled those of the whole study population. (5) Pneumonia cases of different etiologies were included in the study, which introduced some heterogeneity. *Cytomegalovirus*, *Candida spp* and *A. fumigatus* pneumonia are difficult to diagnose unless invasive methods not reported in the present study are used. Moreover, the comparison with biomarkers not expected to increase in the presence of viral or fungal infections is a major limitation. Therefore, to increase practical implications, we performed a *post hoc* analysis limited to proven bacterial infections that confirmed the diagnostic accuracy of PTX3 levels in BAL fluid. (6) In a recent study, researchers showed that receipt of hematopoietic stem cell transplantation from a donor with a homozygous defective expression of PTX3 in neutrophils was associated with an increased risk of invasive aspergillosis [35]. Thus, alveolar PTX3 levels <1 ng/ml warrant caution in their interpretation in patients who raise high clinical suspicion for invasive aspergillosis.

## Conclusions

Our study shows that, in a convenience sample of unselected intubated critically ill patients undergoing BAL, alveolar PTX3 level is discriminative for microbiologically confirmed lung infection. In particular, PTX3 levels ≥1 ng/ml in BAL fluid were associated with elevated sensitivity and NPV, which may enable timely and accurate recognition of the vast majority of true negative cases. Future studies designed to validate the PTX3 threshold and further investigate the role of PTX3 levels in BAL fluid to guide antimicrobial therapy seem warranted.

## Key messages

- Early diagnosis of pneumonia in intubated ICU patients is fundamental but rather challenging, as clinical criteria yield many false-positive results.
- Pentraxin 3 is an acute-phase inflammatory mediator involved in the local recognition and clearance of bacteria, viruses and fungi.
- In the present study, Pentraxin 3 proved to be a useful early marker of pneumonia, with high negative predictive value, in intubated ICU patients.
- A discriminative threshold PTX3 level ≥1 ng/ml in BAL fluid was identified to help translation into clinical practice, but validation is still required.

## Additional file

Additional file 1:
**Additional details regarding methods and results are provided.** The file also contains additional references, one table on BAL fluid microbiology results and one figure illustrating the diagnostic accuracy of PTX3 as a marker of confirmed bacterial pneumonia.

## Abbreviations

AUC^ROC^: Area under the receiving operating characteristic curve
BAL: Bronchoalveolar lavage
CPIS: Clinical Pulmonary Infection Score
CRP: C-reactive protein
NPV: Negative predictive value
PCT: Procalcitonin
PPV: Positive predictive value
PTX3: Pentraxin 3
sTREM-1: Soluble triggering receptor expressed on myeloid cells 1
