# Supplementary material for: Alveolar pentraxin 3 as an early marker of microbiologically confirmed pneumonia: a threshold-finding prospective observational study
Source: Crit Care. 2014 Oct 15;18(5):562. doi: 10.1186/s13054-014-0562-5 (PMC4219103; doi:10.1186/s13054-014-0562-5)

**Alveolar Pentraxin 3 as an early marker of microbiologically confirmed pneumonia: a threshold-finding prospective observational study**

Tommaso Mauri, Andrea Coppadoro, Michela Bombino, Giacomo Bellani, Vanessa Zambelli, Carla Fornari, Lorenzo Berra, Edward A Bittner, Ulrich Schmidt, Marina Sironi, Barbara Bottazzi, Paolo Brambilla, Alberto Mantovani, Antonio Pesenti.

**Additional file 1**

## METHODS

**BAL sTREM-1 assay.** Human sTREM-1 was measured in duplicate with a commercial sandwich ELISA kit (detection limit 8 pg/mL; SIGMA Aldrich, St. Louis, MO; Catalog Number RAB0501) validated for human plasma and serum but not for BAL. To validate the assay for BAL, a spike of sTREM-1 was added to 14 different BAL samples and recovery was evaluated. A recovery of 70-100% was observed in 65% of the samples analyzed while in 20% of samples we had a 50% recovery. In 1 sample out of 14 we were not able to measure the spike of sTREM-1. Thus, these data suggest that the assay used in the present study can measure sTREM-1 levels in BAL with acceptable accuracy.

**Data collection at BAL sampling.** Right before BAL was performed, we recorded: SAPS II score at admission [E1], hospital admission and intubation dates, mechanical ventilation settings, oxygenation, signs of sepsis, sequential organ failure assessment (SOFA) score values [E2], number of organ failures, immunosuppression, clinical pulmonary infection score (CPIS) values [E3] and reason for performing BAL (e.g., pneumonia suspicion or to exclude lung cancer).

**Intra-cellular staining.** The BAL fluids of 20 consecutive patients (all enrolled in Monza) were centrifuged (1500 rpm for 10 min at 4°C) and the cells pellets were re-suspended with 0.5 ml phosphate buffered saline and used for cell counting (Turk solution and Burker chamber). Cells were then spun with Cytocentrifuge (900 rpm for 5 min at 4°C) on different slides to perform PTX3 immuno-staining and evaluate the fraction of BAL cells positive to intracellular PTX3, as previously described [E4]. Briefly, cells were fixed with paraformaldehyde 4%, permeabilized with glycine and incubated with rabbit pAb anti-hPTX3 and then with goat anti-rabbit IgG Alexa Fluor 488 conjugated, while bisbenzimide was used to stain DNA and recognize nuclei.

## RESULTS

**Microbiology results.** Considering all 82 BAL, on the day they were performed, antimicrobial therapy was ongoing in 73 cases (89%). Table E1 reports detailed microbiology results of all culture-positive BAL cases. Positive bacterial cases presenting fungal or bacterial contaminants were 4 (5% of all BALs). Negative cases presenting fungal or bacterial contaminants were 11 (13%), while viral contamination was detected in 16 negative BALfs (19%), likely indicating redundant requests.

**Alternative diagnoses.** Considering all 82 BAL, non-pneumonia cases (n=58, 71%) were classified as follows: BAL performed in patients without clinical signs of pneumonia (n=12, 21%) acute respiratory distress syndrome (ARDS) of extra-pulmonary origin in 17 cases (29%), cardiogenic pulmonary edema in 10 (17%), primary non-infectious ARDS in 10 (17%), acute exacerbations of chronic obstructive pulmonary disease in 3 (5%), lobar atelectasis in 3 (5%) and undetermined in 3 (5%).

**BAL PTX3 in different pneumonia subtypes.** BAL PTX3 levels were increased in presence of VAP in comparison to CAP or HAP (26.26 [5.61-48.59] vs. 2.18 [1.03-7.39] 1.40 [0.00-9.24] ng/mL, $p < 0.05$) likely reflecting clinical severity, while alveolar PTX3 didn't differ between bacterial, viral and fungal etiology ($p > 0.05$, data not shown). When we considered net reclassification index values of BAL PTX3 vs. all other biomarkers and CPIS only for patients with bacterial pneumonia, they were positive (range 0.22-0.51) with gain in sensitivity, specificity or both.

**Correlations between biomarkers.** BAL and plasma PTX3 concentrations were not correlated (rho=-0.068, p>0.05). We couldn't detect any relation between BAL PTX3 and BAL sTREM-1 levels and between BAL PTX3 and circulating sTREM-1, CRP or PCT (p>0.05, data not shown).

**Immuno-stained subset.** In the 20 samples that underwent immuno-staining, BAL PTX3 was significantly higher in pneumonia samples (n=6, 30%) in comparison to negative ones ($p<0.01$, data not shown): thus, this subset might be regarded as representative of the overall population.

## REFERENCES


E1. Le Gall JR, Lemeshow S, Saulnier F: **A new Simplified Acute Physiology Score (SAPS II) based on a European/North American multicenter study.** *JAMA* 1993, **270**:2957-2963.

E2. Vincent JL, Moreno R, Takala J, Willatts S, De Mendonça A, Bruining H, Reinhart CK, Suter PM, Thijs LG. **The SOFA (Sepsis-related Organ Failure Assessment) score to describe organ dysfunction/failure. On behalf of the Working Group on Sepsis-Related Problems of the European Society of Intensive Care Medicine.** *Intensive Care Med* 1996, **22**:707-710.

E3. Pugin J, Auckenthaler R, Mili N, Janssens JP, Lew PD, Suter PM: **Diagnosis of ventilator-associated pneumonia by bacteriologic analysis of bronchoscopic and nonbronchoscopic ‘‘blind’’ bronchoalveolar lavage fluid.** *Am Rev Respir Dis* 1991, **143**:1121-1129.

E4. Jaillon S, Peri G, Delneste Y, Frémaux I, Doni A, Moalli F, Garlanda C, Romani L, Gascan H, Bellocchio S, Bozza S, Cassatella MA, Jeannin P, Mantovani A: **The humoral pattern recognition receptor PTX3 is stored in neutrophil granules and localizes in extracellular traps.** *J Exp Med* 2007, **204**:793-804.

## TABLES

### Table E1. Detailed brocho-alveolar lavage (BAL) microbiology results of patients with pneumonia in the study cohort

| Microbe* | Positive BAL samples† Derivation cohort n=24 |
|---|---|
| ***MRSA*** | 5 |
| ***Streptococcus Pneumoniae*** | 1 |
| ***Pseudomonas Aeruginosa*** | 2 |
| ***Acinetobacter Baumanii*** | 1 |
| ***Enterobacter spp*** | 2 |
| ***Haemophilus Influenzae*** | 2 |
| ***Stenotrophomonas Maltophilia*** | 2 |
| ***Escherichia Coli*** | 1 |
| ***Candida spp*** | 6 |
| ***Aspergillus Fumigatus*** | 3 |
| ***Citomegalovirus*** | 3 |
| ***Herpes Simplex Virus*** | 3 |
| ***Epstein-Barr Virus*** | 2 |
| ***H1N1 Virus*** | 1 |

* MRSA: Methicillin-resistant Staphylococcus aureus.

† Sum exceeds the number of positive BAL cases reported in the text because 10 samples presented growth of multiple non-contaminant microbes.

## FIGURES

**Figure E1. PTX3 as a marker of confirmed bacterial pneumonia.** When we compared only the fifty-eight negative cases vs. the twelve patients with proven bacterial pneumonia (total n=70), PTX3 diagnostic accuracy was confirmed by ROC curve analysis ($AUC^{ROC}$ 0.827, IC95% 0.668-0.987, p<0.0001) while BAL sTREM-1, plasma PTX3, sTREM-1, CRP and PCT couldn’t predict bacterial pneumonia.

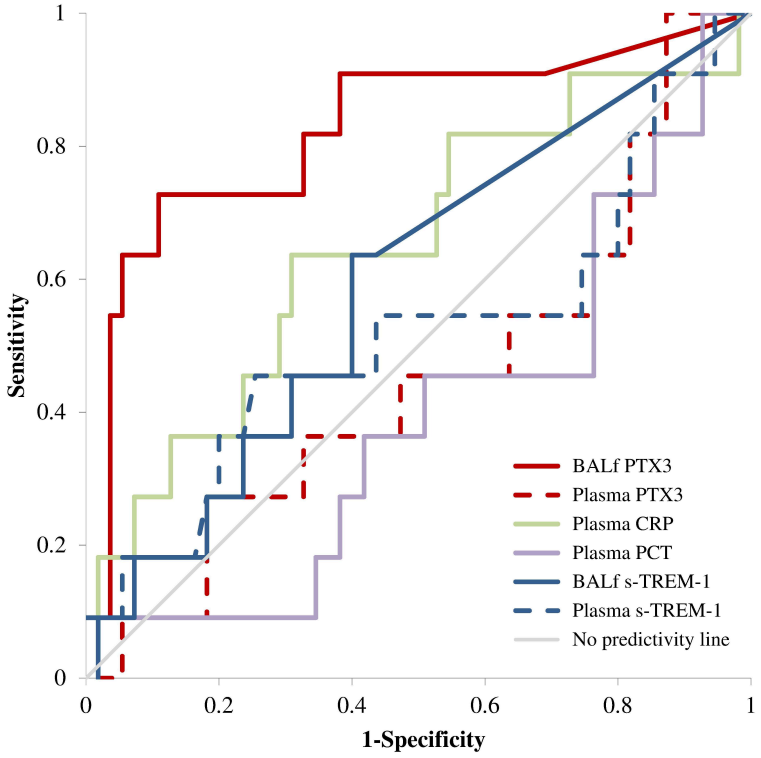

Supplement: Additional file 1: — Additional details regarding methods and results are provided. The file also contains additional references, one table on BAL fluid microbiology results and one figure illustrating the diagnostic accuracy of PTX3 as a marker of confirmed bacterial pneumonia. [file 13054_2014_562_MOESM1_ESM.pdf]
